# Social Structure of Sheep Flocks at Points of the Production Cycle and Relationship to Disease Spread, Using a Simulated Epidemic of Footrot

**DOI:** 10.3390/ani16040587

**Published:** 2026-02-12

**Authors:** Katharine Eleanor Lewis, Emily Price, Darren Croft, Joss Langford, Laura Ozella, Ciro Cattuto, Rachel Clifton, Laura Green

**Affiliations:** 1 School of Life Sciences, University of Warwick, Coventry CV4 7AL, UK; kate.lewis@nottingham.ac.uk; 2 Department of Veterinary Medicine & Science, University of Nottingham, Loughborough LE12 5RD, UK; 3 Centre for Research in Animal Behaviour, University of Exeter, Exeter EX4 4QG, UK; 4 Activinsights Ltd., Cambridge PE28 0LF, UK; 5 Department of Veterinary Sciences, University of Turin, 10095 Grugliasco, TO, Italy; 6 ISI Foundation, 10123 Turin, TO, Italy; 7 Department of Computer Science, University of Turin, 10124 Turin, TO, Italy; 8 Institute of Microbiology and Infection, University of Birmingham, Birmingham B15 2TT, UK

**Keywords:** footrot, lameness, sheep, animal social networks, sheep social networks, disease simulation

## Abstract

Footrot is one of the top five globally important diseases of sheep and causes lameness, leading to poor welfare and productivity. Transmission of *Dichelobacter nodosus*, the causative agent, occurs via surfaces such as pasture or bedding and persistence occurs from diseased sheep shedding bacteria into the environment; *D. nodosus* cannot replicate off host. High resolution proximity sensors were deployed on a flock of Poll Dorset sheep for 10–17 days at several points of the production cycle (teasing, tupping, pregnancy, and lactation (<6-week-old lambs)) between July 2018 and May 2021. Association indices between pairs of sheep were calculated, and outbreaks of footrot were simulated using a network-based susceptible-exposed-infected-recovered model. Two management approaches were modelled (1) where sheep were treated either not promptly, or effectively, resulting in long recovery times (28–100 days) and (2) where sheep were treated and recovered within 15 days, assuming ‘active management’ of footrot by the farmer using ‘best practice’ of prompt recognition of lame sheep and parenteral and topical antibiotics. Under ‘active management’ conditions (scenario 2), outbreak sizes were smaller at all points of the production cycle. This adds to existing evidence that prompt, effective treatment of sheep at all stages of the production cycle is key to reducing the prevalence of footrot in the flock, including at breeding when sheep are more likely to be in close contact.

## Simple Summary

Footrot is an infectious disease of sheep that causes lameness, reducing sheep welfare and costing farmers in terms of both costs of treatment and reduced productivity. While transmission of the causative agent, *Dichelobacter nodosus*, between sheep is likely indirect and occurs via infected pasture, little work has explored how heterogenous connections made by sheep within farm management groups are associated with disease transmission. The aim of this research was to simulate the spread of footrot on real-life social structures of sheep measured using high-resolution social contact data collected at several points of the production cycle: teasing, breeding, gestation, and lactation. We simulated two management scenarios reflecting different types of lameness management, (1) where sheep were treated either not promptly, or effectively, resulting in long recovery times and presence of chronically infected sheep (28–100 days) and (2) where sheep were treated and recovered within 15 days, assuming ‘active management’ of footrot by the farmer using ‘best practice’ of prompt recognition of lame sheep and parenteral and topical antibiotics. We show that the social structure of the flock varies both day to day and over the production cycle and that ‘active management’ of lameness results in smaller outbreak sizes at all stages of the production cycle, adding to the evidence base of the importance of ‘active management’ in reducing lameness levels in sheep flocks.

## 1. Introduction

Heterogenous social connections can influence the spread of disease within groups [1,2] but there has been little exploration of how heterogenous social structures that occur in commercial sheep flocks are associated with disease transmission. Social contact in sheep is mostly based on spatial proximity [3], and detailed sheep social structures can be identified using high-resolution biologging technology such as proximity sensing [4,5]. Farmed sheep live in groups according to stage of the production cycle, and within these groups, sheep tend to associate more with family members and those of similar ages and they also associate based on personality factors [4,5,6,7]. Typically, in U.K. flocks, sheep are bred in October, and lambs are born in March–April, where they spend around 12–16 weeks with their mother before weaning.

One of the top five globally important diseases affecting sheep is footrot. Footrot is an infectious bacterial disease that causes lesions on feet, and consequently lameness. The causal agent is *Dichelobacter nodosus*, an obligate pathogen that persists on feet with clinical signs of footrot; it cannot replicate off-host [8,9]. Transmission of *D. nodosus* occurs indirectly between sheep via pasture/bedding, surviving for minutes to days in the environment transiently in the environment before dying, and for longer in moist environments [8,9,10], and wet weather can result in permanently contaminated pasture [8].

The exact transmission rates of *D. nodosus* between sheep are unknown, but some cases of footrot are associated with spatial proximity to diseased sheep [11], and the most influential parameters associated with the prevalence of lameness in sheep in a simulation study [12] were the rate at which sheep become infected as a result of encountering bacteria in the environment and the bacterial death rate. Since social contact in sheep is strongly associated with spatial proximity [3], it is possible that non-random mixing patterns of association between sheep lead to different bacterial transmission probabilities between animals because of the different likelihoods of coming into contact with contaminated pasture as a result of the spatial–social dynamic.

Network models enable non-random mixing of animals to be investigated (e.g., Weiss et al., modelled the transmission of cetacean morbillivirus in killer whales using a network model). Non-random mixing of animals can have different effects on disease spread, for example, in societies where there are strong sub-grouping effects, disease can become “trapped” in these sub-groups, which occurs in pneumonia outbreaks in wild bighorn sheep [13,14]. The aim of this paper was to examine how different social structures that occur through the production cycle within a flock of Poll Dorset sheep could influence the transmission of footrot at different points of the production cycle.

## 2. Materials and Methods

Data collection was approved by the University of Exeter’s ethical board (reference number: eCLESPsy000541, date of approval: 25 June 2018).

### 2.1. Sheep and Study Environment

The study was carried out on a commercial farm of Poll Dorset sheep in the UK. The production cycle was typical for Poll Dorsets, which are unusual in that they are able to breed all year. The breeding cycle starts in mid-March, when oestrous is stimulated by introducing vasectomised rams to a group of ewes for four weeks. Following this, one fertile ram is introduced to a sub-group of ewes for two oestrous cycles (five weeks). Pregnant ewes then live in a single flock until lambs are born in September–October. Two deployments of sensors were carried out during gestation, one when lambs were at foot, one with teasers, and three during breeding; the dates and details of these deployments are listed in Table 1. Summaries of the weather conditions and space available to the sheep over each deployment are in Figure S1 and Table S1.

### 2.2. The Proximity Sensing Platform and Data Processing

Contacts between sheep were recorded using proximity sensors designed and developed by the SocioPatterns collaboration (http://www.sociopatterns.org), which exchange low-power radio packets that allow for the recording of close contacts of 1.0–1.5 m, approximately the body length of an adult sheep. The hardware is open-source and based on the design developed by the OpenBeacon project (http://www.openbeacon.org); a more detailed explanation of the sensing system can be found in [5]. The sensors sampled at a 20 s temporal resolution, and sheep were defined as associated when the sensors exchanged at least one radio packet within the 20 s time interval. Contacts between sheep were excluded if the number of radio packets exchanged and received was not recorded in a symmetric way, or if the sensor did not make any contacts over the study period. When ewes had young lambs at foot, data from the whole family group (a dam and her lamb(s)) were excluded if one sheep in the family needed to be excluded.

Contacts were aggregated on both a daily basis (midnight–midnight), since sheep have consistent diurnal patterns [17] and sleep transiently [18], and over the whole study period. Details of the contacts collected within each deployment of the sensing system are in Table 2.

### 2.3. Social Network Creation

All analysis was carried out in R Version 4.2.0 [19] using the *tidyverse* for data manipulation [20]. Time-aggregated weighted networks were generated from the contact data. The nodes represent each sheep and edges correspond to the dyadic association index, which is the probability that sheep_a_ and sheep_b_ associate within a period of time, calculated as follows:

$$AI=\frac{x_{ab}}{x_{ab}+x_{a}+x_{b}}$$

The association index (AI) equals the number of sampling periods (the 20-s temporal window detected by the proximity sensors), with individual *a* and individual *b* associated divided by the number of sampling periods where *a* and *b* were associated, plus the number of sampling periods where individual *a* was detected without *b*, and where *b* was detected without *a*. The index ranges from 0 (the two individuals were never observed together) to 1 (the individuals were always observed together).

Association networks were calculated per day and over the whole deployment. We were interested in both time scales; the daily scale since health status can change on a daily basis, and the whole period of sensor deployment, as these correspond approximately to the incubation period of footrot, which is between 8 and 14 days [21,22].

### 2.4. Social Network Analysis

#### Local Cosine Similarity

To assess the similarity of the associations made between sheep within a production group on a day-to-day basis, social stability was calculated using Local Cosine Similarity (LCS). LCS is a measure of similarity that looks at ego-networks, the sheep to which an individual sheep is directly connected at two time points. The LCS for sheep_a_ is given by calculation of the cosine similarity between vectors of weights of the association indices involving sheep_a_ in each network.

$$LCS\left(i\right)=\frac{\sum_{b}{AI}_{ab}^{\left(t1\right)}{AI}_{ab}^{\left(t2\right)}}{\sqrt{\sum_{b}{\left({AI}_{ab}^{\left(t1\right)}\right)}^{2}}\sqrt{\sum_{j}{\left({AI}_{ab}^{\left(t2\right)}\right)}^{2}}}$$

where ${AI}_{ab}^{\left(t1\right)}$ is the association index between sheep_a_ and sheep_b_ at time point 1, and ${AI}_{ab}^{\left(t2\right)}$ is the association index between sheep_a_ and sheep_b_ at time point 2. The value ranges from 0 to 1, and the higher the value, the more similar the associations of the individual at time point 1 and time point 2.

Global network stability was calculated as the mean value of all the individual sheep’s LCS. Temporal evolution of the network was calculated via the LCS of each day (t) and the next day (t + 1).

### 2.5. Formation of the SEIR Model

A stochastic individual-based susceptible-exposed-infected-recovered (SEIR) model was used to simulate the spread of footrot over the social networks at each stage of the production. A SEIR model is a simplistic assumption for footrot spread because sheep are able to recover and become re-diseased [23]; although there is some short-term immunity to footrot, for some sheep, there is no immunity but for others it can last 4–6 months. We were interested in transmission over short time scales and the time that sheep would spend in different groups at each stage of the production cycle, and we assumed 150 days as an average for this, with 10,000 simulations each time, and assumed that sheep would be unlikely to be re-infected within this period. In practice, the time spent in a group varies by the stage of production (e.g., time in mating groups is usually shorter than gestation).

Epidemiological parameters for footrot have previously been estimated [10,21,23,24,25] and used in a simulation model [12]. For our model (Table 3), the transmission parameter (β) was simulated from a range of 0.0001–0.1, the rate of conversion from a latent to infectious state was 2–10 days, and we examined two rates of recovery α_1_, 28–100 days, representing a situation where sheep were not treated promptly and chronically diseased sheep remained in the field, and α_2_, 3–15 days, representing a situation where sheep were treated promptly with an effective treatment. Our model assumes that all transmission occurs from infected sheep being a proxy for spread via infected ground.

For each set of simulations, β, α, Σ and seeded individual were drawn via Latin hypercube sampling using *lhs* [26].

The simulation represented one infectious individual entering the production group. Although footrot is endemic in most flocks in England [27,28], it is possible for this to occur in commercial settings, for example, from buying in sheep with footrot, or introducing a diseased ram into a group of uninfected ewes at mating.

The model followed the format of Weiss et al. (2020) [29]—the probability that individual *j* transmits the disease to susceptible individual *i* at time t (λ_tab_) is modelled as the joint probability that sheep *a* and *b* come into contact and transmit disease. For diseases with long incubation periods, considering the probability of contact over longer periods of time is important [30]; since the incubation period of footrot (7–14 days [21]) is long relative to the deployment periods of approximately two weeks, we used the AI indices over the whole study period for the contact as our probability of contact. This probability that sheep *a* and *b* come into contact and transmit disease was modelled as:

$$\lambda_{tij}=\beta *{AI}_{ab}*I_{tb}$$

where β is the transmission coefficient, ${AI}_{ab}$ is the overall association index between individuals *i* and *j* over each deployment period, and $I_{tb}$ is an indicator variable that is 1 if the individual *a* is infectious and 0 if not. The probability that the susceptible individual becomes infected (but not yet infectious) during timestep t (T_ta_) is

$$T_{ta}=1-\prod_{b}(1-\lambda_{tab})$$

Individuals that are infected at time t do not become infectious or recover until t + 1. The probability that individuals become infectious at t + 1 is determined by ς, and that they recover is determined by α (mean infectious period = 1/α). The model run is terminated when there are no infected individuals left, or the time limit is reached.

We were interested in two situations regarding recovery rates:

1. Sheep have low recovery rates of 28–100 days because farmers are slow to treat lame sheep or use ineffective treatments [27,28].
2. Sheep are able recover within 15 days because farmers treat all lame sheep immediately with an effective treatment such as parenteral antibiotics [25].

Simulations were assessed using the mean outbreak size, and proportions of diseased sheep of interest were taken from:

- <2%—the Farm and Animal Welfare Council target for the national prevalence of lameness by 2021 [31].
- >5%—sheep farmers in England consider a prevalence of 5–7.5% lameness acceptable.
- >10%—English sheep farmers consider >10% lame sheep in a flock unacceptable [32].

To assess the effect of social structure on the outbreak size, two null models were tested:

- Mean-field association network, where all individuals had the same connection to each other—the mean of all the association indices in the deployment, which simulates a traditional “random mixing” model.
- Edge-permuted network, where the edge weights are randomly shuffled between individuals, retaining the heterogeneity of social preferences but removing the higher order structure of the network.

The sensitivity of the models to the input parameters (β, α, and ς) was assessed using partial correlation coefficients with *ppcor* [33].

## 3. Results

### 3.1. Association Indices Between Sheep

**Figure 1:**
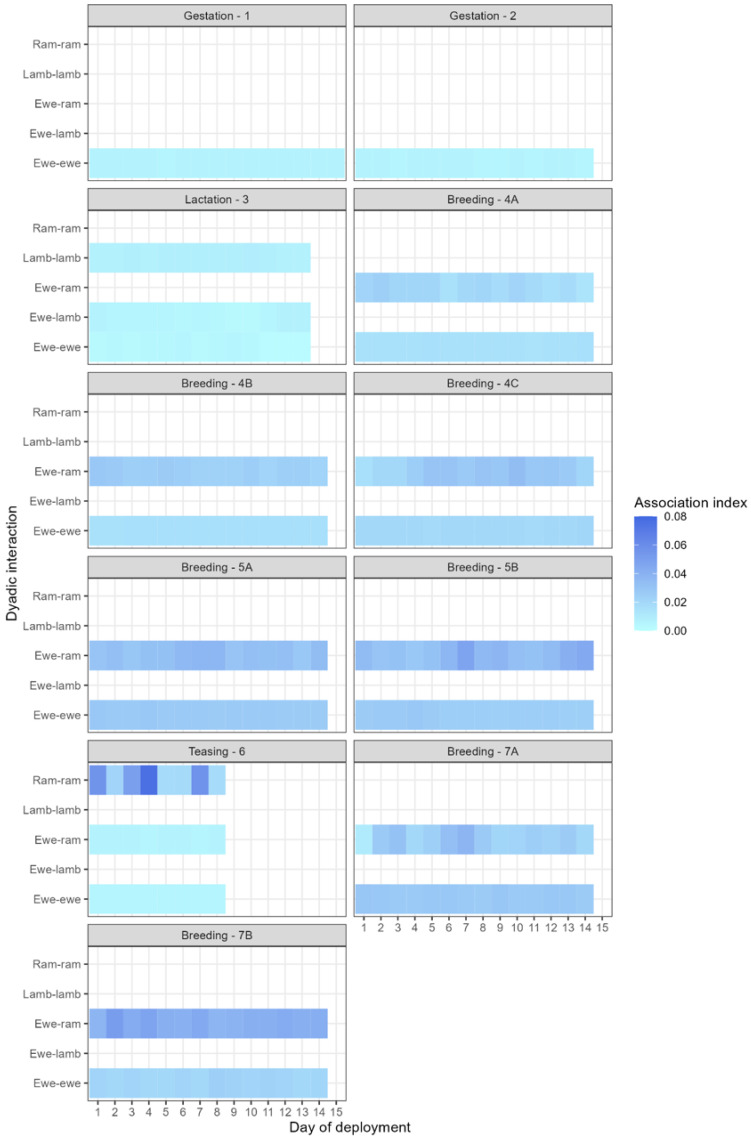
Mean daily association indices for the different dyad types within each stage of the production cycle (gestation, lactation, teasing, and breeding).

Ewes associated with each other in each deployment, regardless of presence of rams or lambs (Table 4). At breeding, ewe–ram associations were similar across each of the tupping groups (Table 4) within the deployment (Table 4), and there was little difference in association rates between seasons (D4 and D7, spring, and D5, summer, see Table 1 for deployment details). Lambs associated with both lambs and ewes; more work on lamb interactions and contacts between and within family groups is available in Ozella et al. (2022) [6] and Lewis et al. (2022) [11]. In each deployment, there was day-to-day variation in mean association rates between the dyad types (Figure 1).

### 3.2. Global Stability of the Network

**Figure 2:**
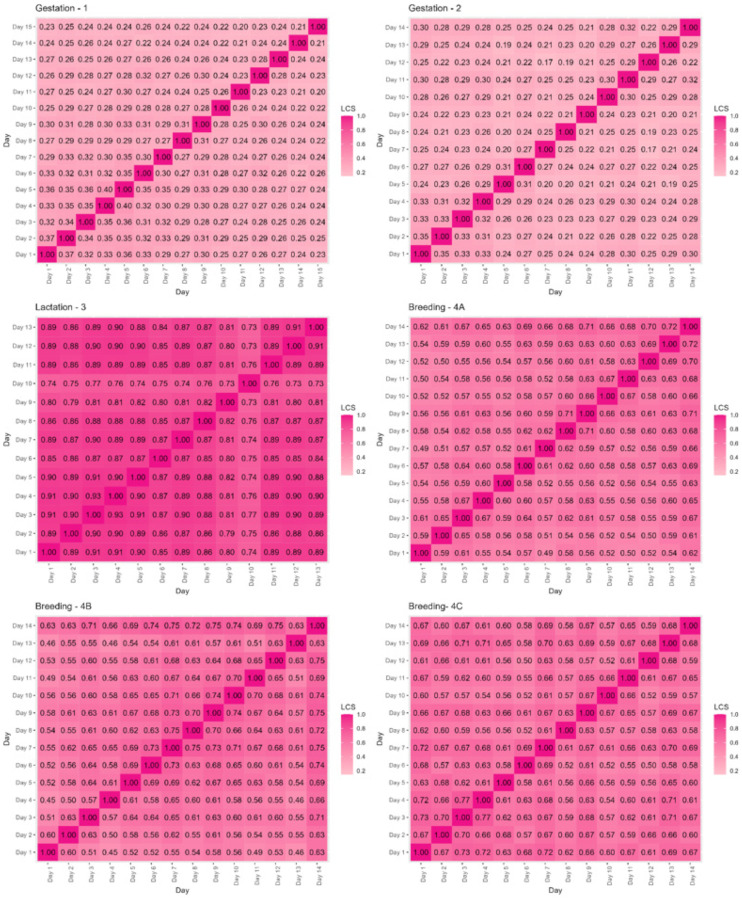

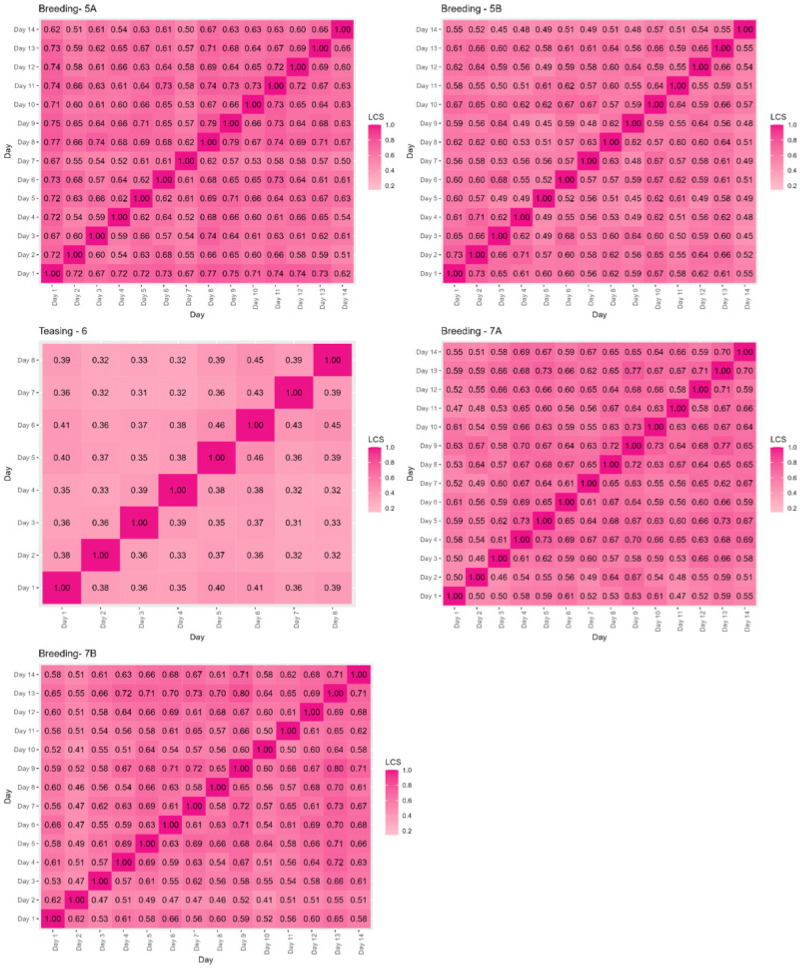
Global network stability indicated by the mean daily local cosine similarity values for each day of deployment by production stage. LCS = global mean daily local cosine similarity.

**Figure 3:**
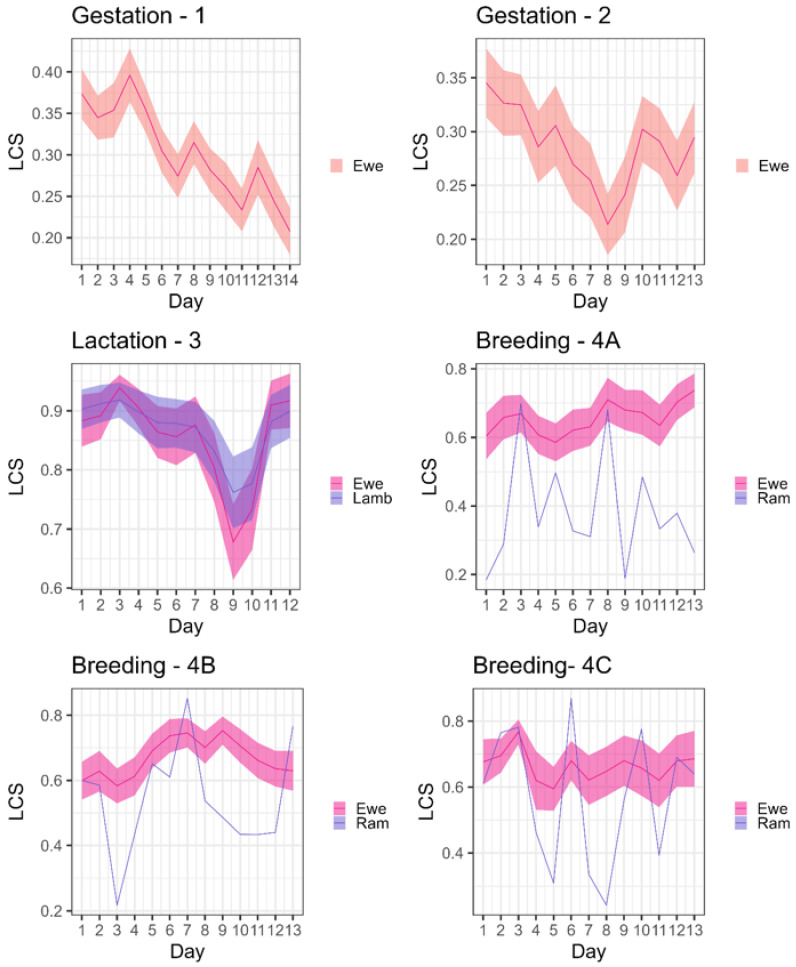

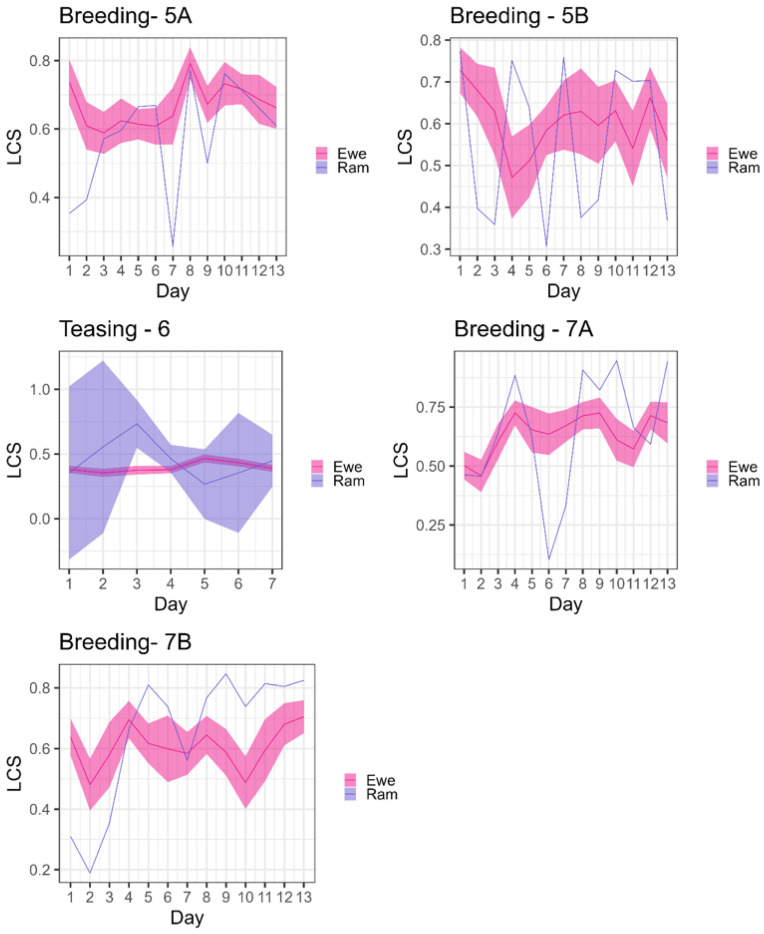
Temporal evolution of ego-networks computed between one day and the next, indicated by local cosine similarities (LCS) between each day and the next (t and t + 1) for each stage of the production cycle. The lines give for each group give the average of cosine similarity value between each day of the deployment and the next, shaded areas show the 95% confidence interval.

Ewes were most flexible in their social patterns when alone at gestation (Figure 2, Supplementary Table S2), with the lowest global mean LCS between each day of the deployment (global mean LCS = 0.26 (D1)–0.29 (D2)), followed by at teasing (global mean daily LCS = 0.38). Social networks were more stable from day to day at breeding (global mean daily LCS = 0.60–0.67 at breeding (D4, D5 and D7) and at lactation, when lambs were at foot (global mean daily LCS = 0.84). The temporal evolution of each social network is shown in Figure 3; at breeding, social stability came from the patterns of ewes, while there was higher variation in social patterns of the rams. Similarly, at teasing, the social patterns of ewes were much more stable (Figure 3) than those of the rams (although there were only three rams in the deployment compared to the 85 ewes).

### 3.3. SEIR Model

The distribution of outbreak sizes was highly right-skewed in both the treatment and non-treatment conditions (Supplementary Figure S2). The mean outbreak sizes were lower in the treatment than non-treatment conditions; in effective treatment conditions, the mean outbreak sizes were <2% in non-breeding deployments and 4–6% in breeding deployments, while under ineffective treatment conditions, the mean outbreak sizes were <5–8% in non-breeding deployments and 14–20% in breeding deployments.

Breeding deployments had higher association indices between sheep (Table 4, Figure 2) than other points of the production cycle; these higher association probabilities likely account for the higher mean outbreak sizes. Within each breeding deployment, the social structure was not protective against outbreaks; mean outbreak sizes and probabilities of obtaining >10% infected sheep on the observed network were similar to those on the two null networks.

At lactation, mean outbreak sizes were low (5% under ineffective treatment, 1% under effective treatment conditions, Table 5), and under ineffective treatment conditions, the observed social structure was protective against outbreaks; the probability of >10% infected sheep was 0.123, while it was higher on the edge-permuted network (0.152) and mean-field network (0.203) (Supplementary Table S3). Under effective treatment conditions, social structure had less protective effect against outbreaks—the probability of >10% infected sheep was 0.001 in the observed network, edge permuted network, and the mean-field network (Table 5, Supplementary Table S3). At lactation, association probabilities between sheep are extremely heterogenous; family groups have much higher probabilities of associating compared with non-family members (in Ozella et al., 2022, Lewis et al., 2022 [6,11]), and footrot is able to spread within family groups as a result of spatial proximity, but not out of them [11], which results in the protective effect of social structure compared with the mean-field and edge-permuted networks (Table 5, Supplementary Table S3).

In the observed networks, the transmission parameter (β) was positively correlated with the final outbreak size at all stages of the production cycle, and the time to recovery parameter (α) was negatively correlated with the final outbreak size (Supplementary Table S4).

## 4. Discussion

Both the social structure and the stability of these social structures within sheep flocks vary with stage of the production cycle, and the social structures that occur as a result of farm management have different potentials for disease transmission. Tupping is a key point at which disease could spread through flocks, because higher probabilities of association between sheep result in higher mean outbreak sizes (Table 5). At all points of the production cycle, faster recovery rates, which are achieved in practice with effective treatment regimes, resulted in lower mean outbreak sizes and probabilities of >10% lame sheep. Having >10% lame sheep is deemed unacceptable by English sheep farmers [32] and this study adds to the existing evidence base that active management of lame sheep to reduce recovery times is essential to achieving low flock prevalence of lameness [25,27], likely because reducing the time sheep are infectious reduces the potential to transmit *D. nodosus* to other sheep in the flock.

### 4.1. Considerations Around Spread of Footrot Within the Flock

Treatment of sheep with footrot with parenteral antibiotics and foot spray within three days of identifying lameness is the most effective treatment currently available and can reduce flock prevalence of lameness to <2% [25,34]. In practice, farmers treat sheep differently at different stages of the production cycle, for example, farmers may not like to treat heavily pregnant ewes, or due to other commitments on the farm [35]. We demonstrate that effective management resulting in fast recovery rates throughout the production cycle is key to obtaining low prevalence of lameness. Farmers would usually have little control over the environmental transmission rates in their flock but are able to lower the number who become infected by having few infected sheep who are shedding bacteria into the environment, and the fast recovery of the ones that do contract footrot; both of which reduce the force of infection as fewer sheep are shedding bacteria into the environment. The exact transmission rate of footrot between sheep is unknown, although it has been identified as important in previous simulation studies [12], and therefore, here, we simulated a range of transmission parameters to reflect varying infection rates that, are in practice, related to unknown environmental effects.

Differences in spread of footrot between types of groups of sheep has previously been observed—spread appears faster within groups of rams compared to ewes [36], and ewes are more likely to become lame if their lambs are lame, and vice versa [11]. One explanation for this is that the type of behaviours observed between rams or ewes and their lambs are behaviours that bring them into closer physical proximity, making sheep more likely to pick up bacteria shed by infected sheep—for example, mounting/butting between rams, or suckling/sucking between ewes and their lambs, whereas physical contacts between ewes when they are alone mostly involve head-rubbing or sniffing [37]. Despite the close proximity of ewes to their lambs, this was the only point of the production cycle where the social structure was protective against large outbreaks compared to the null networks (Table 5, Supplementary Table S3). Significant sub-grouping of the sheep occurred at this point of the production cycle, as family groups have much higher probabilities of associating than non-family sheep [6,11], and sub-grouping can be protective against disease spread [14,38]. Here, disease likely spreads easily between ewes and their lambs, but not out of family groups, as connections between family groups are much weaker than within family groups [11]. We note here that our simulation represents one infected sheep entering the production group; if multiple sheep are lame when a management group is created, this is likely to mean that disease spreads faster throughout the group, which is why evidence-based management involves treatment of the first lame sheep in a group [27,34].

### 4.2. Considerations Around Environmental, Social, and Data-Processing Influences on Transmission

Since the sheep production cycle is intrinsically linked to the time of year, it is difficult to separate social effects from environmental effects as social behaviour in sheep is influenced by the environment, as sheep spend more time together when it is wet or cold [4,6], or when new grass is available on the strip grazing system [5]. The environment also influences footrot transmission; higher stocking densities are associated with higher prevalence of lameness [39], and wet conditions pre-dispose the feet to infection [40]. In the U.K., farmers report that prevalence of lameness is uneven over the year, with sheep lame in certain months more than others [41]. The most obvious explanation for this is that differing environmental conditions in different months create different susceptibilities of the hoof to infection, along with different bacterial survival rates in the environment, and a combination of these factors leads to different transmission rates. It is also possible that the increased time spent in close proximity to other sheep when it is wetter contributes to the increased likelihood of uninfected sheep acquiring bacteria as a result of spatial proximity to infected sheep. Spatial proximity to infected sheep accounts for a small proportion of cases of footrot in the flock [11]. Physiological stage of the gestation also influences immune responsiveness [42,43], which could affect disease development; it is difficult to fully untangle associations between environmental, physiological, and social effects.

Transmission on empirical dynamic contact networks is influenced by data processing decisions and should be guided by the pathogen of interest [30]. Data aggregated over short time windows are more likely to be informative for modelling the transmission of infections with short infectious periods, while aggregation over longer periods is more appropriate for those with longer infectious periods [44]. When contacts are aggregated, this can affect transmission chains—for example, if A meets B, and B is infected, then A meets C, then disease can be transmitted from B to A or A to C, but if A met C before B, this is no longer possible [45]. Since the incubation period for footrot is 7–14 days and our contact data spanned approximately 2 weeks in each deployment, the aggregated association indices were used in the SEIR model.

### 4.3. Limitations of the Current Work

One limitation of this work is that it assumes the probability that sheep *a* transmits the disease to susceptible sheep *b* at time *t* is the joint probability that sheep *a* and *b* come into contact and transmit disease, and all transmission occurs as a result of this joint probability. We did not solely model environmental transmission because even though in practice *D. nodosus* is able to survive in soil, it cannot survive in the absence of infected sheep. For almost all farmers, the production cycle and sheep behaviour is intrinsically linked to the time of year, meaning that the joint probability of transmission was of particular interest here because in practice, it is not possible to separate the effects of these. We also only examined the emergent social structures that occurred as a result of the environment that the sheep were kept in, and we did not have full information on some of the parameters that influence these, such as stocking density, which will influence the association rates between animals. We also did not consider the impact of farm management practices (e.g., in practice on this farm, ewes and lambs are brought in for 24 h post lambing, and these effects of ‘missing’ sheep are not currently modelled in any deployment). As biologging technology continues to improve (longer battery life, lighter sensors, and improved memory), longer studies will become possible, which would better reflect the endemic nature of footrot in U.K. sheep flocks and better capture the interaction of the environment and weather conditions that are known to affect both footrot prevalence [8,40] and sheep behaviour [4,5].

### 4.4. Contribution to Future Control of Footrot

This work highlights the importance of the active management of sheep throughout the stages of the production cycle. While the ability of farmers to treat sheep over the production cycle varies due to farm and individual specific factors [35], reducing the time that sheep are lame, and therefore likely shedding bacteria into the environment to be encountered by other sheep, is key to reducing flock prevalence of lameness. Here, we show that social structures within production groups influence outbreak sizes. Combined with the knowledge that footrot transmits more easily within family groups than out of them at lactation when lambs are very young [11], we suggest non-lame sheep within family groups should be monitored so that they can be treated quickly to minimise the effect of lameness on their health and welfare, as these sheep are at high-risk of becoming lame.

## 5. Conclusions

This work provides insight into the social structure of sheep flocks at different points of the production cycle in a commercial flock. Fast recovery of sheep with footrot is key to maintaining <2% lame sheep at all stages of the production cycle, and tupping may be a key point where ewes become infected with footrot, as sheep have a higher probability of associating with each other when in small tupping groups. We highlight the importance of the active management of lame sheep at all stages of the production cycle to prevent spread between sheep because faster recovery rates result in reduced opportunity for transmission of disease between sheep, overall reducing the size of disease outbreaks.

## Figures and Tables

**Table 1:** Reference table showing the production cycle stage, deployment label, season, dates of the deployment, and existing work involving each dataset.

Production Cycle Stage	Deployment	Sheep	Season	Date (from–to)	Further Information
Gestation	1	Ewes	Summer	2–18 July 2018	Ozella et al., 2020 [5]
Gestation	2	Ewes	Summer	2–18 August 2018	Ozella et al., 2020 [5]
Lactation	3	Ewes	Autumn	1–15 October 2019	Ozella et al., 2022 [6]
					Lewis et al., 2022 [11]
		Lambs			Price et al., 2022 [15]
					Lewis et al., 2023 [16]
Breeding	4 (4A, 4B, 4C)	Ewes	Spring	17 April 2020–2 May 2020	
		Rams (tups)			
Breeding	5 (5A, 5B)	Ewes	Summer	5–20 July 2020	
		Rams (tups)			
Teasing	6	Ewes	Spring	10–20 April 2021	
		Rams (teasers)			
Breeding	7 (7A, 7B)	Ewes	Spring	11–26 May 2021	
		Rams (tups)			

Where a letter is indicated after the deployment number, this indicates there were multiple groups of sheep.

**Table 2:** Production cycle stage, number of sheep in the group, the number of sheep with complete contact data for the deployment, and the number of contacts recorded.

Production Cycle Stage	Sheep	Number of Midnight-Midnight Periods	Number of Sheep in Field	Number of Sheep with Complete Contact Data	Number of Contacts Recorded
Gestation—1	Ewes	15	86	84	146,861
Gestation—2	Ewes	14	86	84	95,618
Lactation—3	Ewes	13	50	40	216,054
	Lambs		68	54	
Breeding—4A	Ewes	14	31	30	87,108
	Rams (tups)		1	1	
Breeding—4B	Ewes	14	30	29	94,923
	Rams (tups)		1	1	
Breeding—4C	Ewes	14	23	22	60,708
	Rams (tups)		1	1	
Breeding—5A	Ewes	14	24	18	52,327
	Rams (tups)		1	1	
Breeding—5A	Ewes	14	24	18	21,336
	Rams (tups)		1	1	
Teasing—6	Ewes	8	88	85	113,902
	Rams (teasers)		3	3	
Breeding—7A	Ewes	8	22	17	14,736
	Rams (tups)		1	1	
Breeding—7B	Ewes	14	23	18	15,969
	Rams (tups)		2	1	

**Table 3:** Parameters tested in the SEIR model.

Parameter	Range	Rationale for Use	Source *
Rate of transmission (β)	0.00001–0.1 *	Simulated	Russel et al., (2013) [12]
Rate of conversion from latent state to infectious state (ς)	0.1–0.5	Average duration = 1 week (0.15)	Egerton et al. (1969) [21]
	(2–10 days)		Roberts and Egerton (1969) [24]
Rate of recovery—ineffective treatment and presence of chronically diseased sheep (α_1_)	0.035–0.01	Average duration = 4 weeks (0.03)	Beveridge (1941) [10], Egerton et al., 1969 [21], Roberts and Egerton (1969) [24] Egerton et al., (1983) [23]
	(28–100 days)		
Rate of recovery—active management of lameness (α_2_)	0.3–0.065	65% of sheep treated with paterental antibiotics recover within 5 days	Kaler et al., (2010) [25]
	(3–15 days)		

* Infection rate values are unknown, so we used the range of values tested in Russel et al. (2013) [12] which were tested on sample model runs to determine values for base and variations.

**Table 4:** Summary of association indices between dyad types over the whole deployment at each stage of production.

Stage of Production Cycle		Association Index				
Deployment	Dyadic Interaction	N	Mean	SD	Min	Max
Gestation—1	Ewe–ewe	3486	0.006	0.004	0.000	0.036
Gestation—2	Ewe–ewe	3486	0.006	0.005	0.000	0.060
Lactation—3	Ewe–ewe	780	0.004	0.003	0.000	0.024
	Ewe–lamb	2160	0.005	0.030	0.000	0.494
	Lamb–lamb	2862	0.008	0.032	0.000	0.472
Breeding—4A	Ewe–ewe	435	0.015	0.011	0.000	0.102
	Ewe–ram	30	0.019	0.011	0.005	0.041
Breeding—4B	Ewe–ewe	406	0.016	0.009	0.000	0.054
	Ewe–ram	29	0.024	0.009	0.009	0.049
Breeding—4C	Ewe–ewe	231	0.020	0.014	0.000	0.065
	Ewe–ram	22	0.026	0.014	0.000	0.049
Breeding—5A	Ewe–ewe	153	0.027	0.009	0.009	0.054
	Ewe–ram	18	0.033	0.012	0.011	0.057
Breeding—5B	Ewe–ewe	153	0.026	0.013	0.006	0.070
	Ewe–ram	18	0.037	0.021	0.017	0.111
Teasing—6	Ewe–ewe	3570	0.005	0.004	0.000	0.033
	Ewe–ram	255	0.006	0.008	0.000	0.079
	Ram–ram	6	0.039	0.028	0.020	0.076
Breeding—7A	Ewe–ewe	136	0.028	0.017	0.004	0.101
	Ewe–ram	17	0.025	0.021	0.005	0.084
Breeding—7B	Ewe–ewe	153	0.022	0.015	0.002	0.071
	Ewe–ram	18	0.042	0.022	0.008	0.084

N = number of possible dyads (pairs of sheep), Mean = arithmetic mean, SD = standard deviation, Min = minimum, Max = maximum.

**Table 5:** Results of 10,000 simulations of the SEIR model on the observed association networks at each stage of the production cycle for 150 days.

	Outbreak Size—Proportion Flock Infected				Probability of Percentage Infected Sheep		
Network	Mean	SD	Min	Max	≥2%	≥5%	≥10%
Ineffective management							
1	0.077	0.138	0.012	0.940	0.431	0.259	0.194
2	0.074	0.133	0.012	0.952	0.433	0.254	0.190
3	0.045	0.077	0.011	0.755	0.454	0.214	0.123
4A	0.141	0.201	0.032	1.000	1.000	0.428	0.274
4B	0.146	0.207	0.033	1.000	1.000	0.431	0.327
4C	0.161	0.210	0.043	0.913	1.000	0.403	0.304
5A	0.202	0.247	0.053	1.000	1.000	1.000	0.440
5B	0.197	0.240	0.053	1.000	1.000	1.000	0.442
6	0.069	0.125	0.011	0.852	0.430	0.246	0.180
7A	0.190	0.228	0.056	1.000	1.000	1.000	0.427
7B	0.171	0.212	0.053	1.000	1.000	1.000	0.408
Active management							
1	0.014	0.008	0.012	0.214	0.086	0.007	0.001
2	0.014	0.008	0.012	0.274	0.082	0.006	0.002
3	0.012	0.006	0.011	0.191	0.089	0.005	0.001
4A	0.037	0.020	0.032	0.581	1.000	0.082	0.011
4B	0.038	0.020	0.033	0.467	1.000	0.082	0.027
4C	0.049	0.025	0.043	0.478	1.000	0.073	0.024
5A	0.060	0.032	0.053	0.579	1.000	1.000	0.090
5B	0.060	0.031	0.053	0.737	1.000	1.000	0.089
6	0.013	0.006	0.011	0.125	0.076	0.005	0.000
7A	0.063	0.030	0.056	0.722	1.000	1.000	0.083
7B	0.059	0.028	0.053	0.684	1.000	1.000	0.079

Mean = mean outbreak size, SD = standard deviation of outbreak sizes, min = minimum, max = maximum.

## Data Availability

The raw data supporting the conclusions of this article may be made available by the authors on request.

## Supplementary Materials

The following supporting information can be downloaded at: https://www.mdpi.com/article/10.3390/ani16040587/s1, Figure S1. Summary of the mean daily temperature-humidity index (°C), wind chill index (°C) and total daily rainfall (cm) measured by the Davis pro-Vantage weather station for each day*; Table S1. Space available (hectares) to the sheep throughout the deployments; Table S2. Overall mean of the mean daily local cosine indexes for point of the production cycle; Figure S2. Distribution of the proportion of diseased sheep infected from the 10,000 simulations of the SEIR model at different points of the production cycle under (a) 28–100 day recovery rates (ineffective treatment) and (b) 3–15 day recovery rates (active management of lameness); Table S3. Results of 10,000 simulations of the SEIR model on the observed, edge permuted and mean-field association networks at each stage of the production cycle, for 150 days; Table S4. Spearman’s partial correlation coefficient for model parameters with the final outbreak size in the SEIR models.

## Abbreviations

The following abbreviations are used in this manuscript:

LCS	Local cosine similarity
SD	Standard deviation
N	Number
Min	Minimum
Max	Maximum
