# Supplementary material for: Social Structure of Sheep Flocks at Points of the Production Cycle and Relationship to Disease Spread, Using a Simulated Epidemic of Footrot
Source: Animals (Basel). 2026 Feb 12;16(4):587. doi: 10.3390/ani16040587 (PMC12937435; doi:10.3390/ani16040587)
Supplement: Supplementary file 1 [file animals-16-00587-s001.zip › animals-4114697-supplementary.pdf]

Supplementary Material

Figure S1. Summary of the mean daily temperature-humidity index (°C), wind chill index (°C) and total daily rainfall (cm) measured by the Davis pro-Vantage weather station for each day*

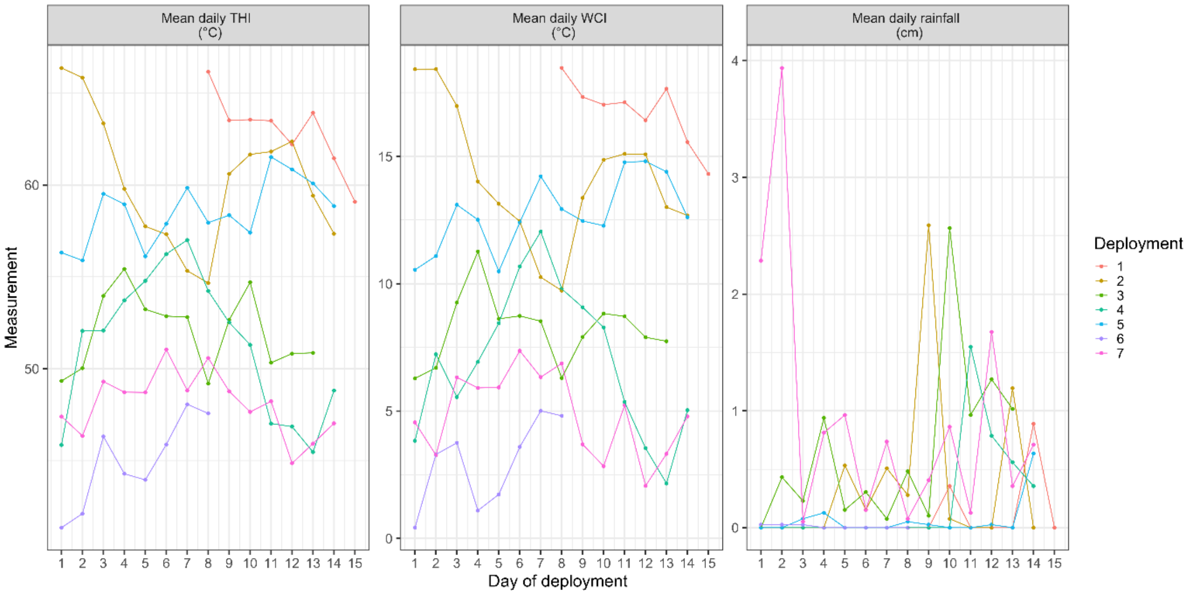

` *7 days of weather data are missing for D1 (03/07/2018-09/07/2018)

Table S1. Space available (hectares) to the sheep throughout the deployments.

Deployment	Field size (ha)	Number of days spent in field	Days of deployment spent in field
1	1.12	5	1-5
	2.27	1	6
	2.03	9	7-15
2	2.47	9	1-9
	1.92	5	10-14
3	0.69	4	1-4
	1.34	4	5-8
	1.98	5	9-13
4A	2.03	13	1-13
	1.94	1	14
4B	1.94	13	1-13
	2.03	1	14
4C	1.18	13	1-13
	1.15	1	14
5A	1.37	14	1-14
5B	1.18	16	1-14
6	2.89	6	1-6
	2.13	2	6-8

7A	2.03	14	1-14
7B	1.91	14	1-14

1. ha = hectare, days of deployment refer to the number of days with midnight-midnight contact data.

Table S2. Overall mean of the mean daily local cosine indexes for point of the production cycle

Deployment	Mean	SD	Min	Max
1	0.29	0.04	0.21	0.41
2	0.26	0.04	0.18	0.37
3	0.84	0.04	0.75	0.91
4A	0.60	0.05	0.50	0.74
4B	0.62	0.07	0.45	0.75
4C	0.63	0.05	0.50	0.77
5A	0.66	0.06	0.52	0.80
5B	0.60	0.05	0.47	0.72
6	0.38	0.04	0.32	0.47
7A	0.65	0.07	0.49	0.77
7B	0.67	0.07	0.51	0.84

1. SD = standard deviation, min = minimum, max = maximum

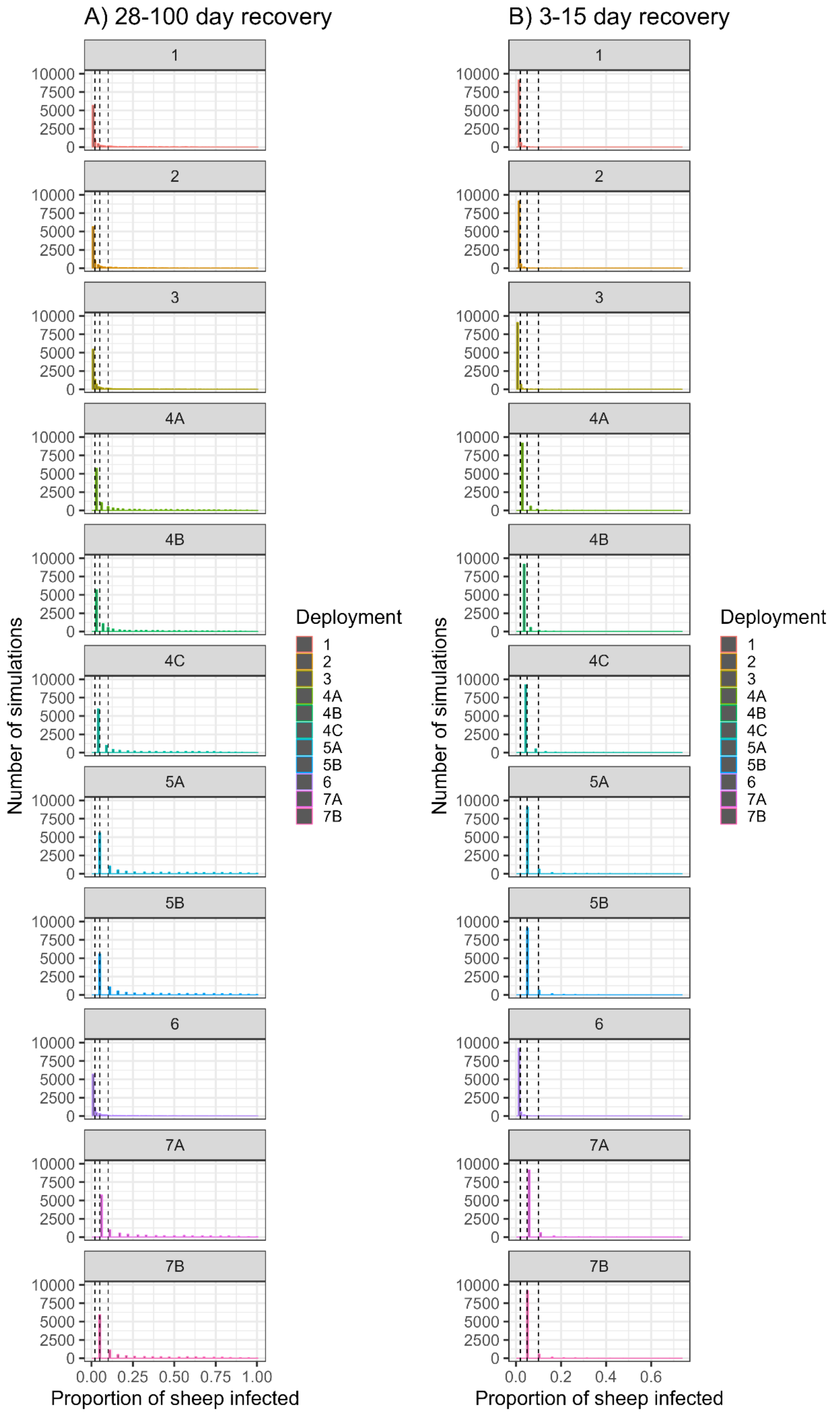

Dashed lines are placed at 2%, 5% and 10% of the flock infected

Figure S2 Distribution of the proportion of diseased sheep infected from the 10000 simulations of the SEIR model at different points of the production cycle under a) 28-100 day recovery rates (ineffective treatment) and b) 3-15 day recovery rates (active management of lameness)

Table S3. Results of 10000 simulations of the SEIR model on the observed, edge permuted and mean-field association networks at each stage of the production cycle, for 150 days

	Outbreak size - proportion flock infected				Probability of percentage infected sheep		
**Network**	**Mean**	**SD**	**Min**	**Max**	**≥2%**	**≥5%**	**≥10%**
Observed (ineffective management)							
1	0.077	0.138	0.012	0.940	0.431	0.259	0.194
2	0.074	0.133	0.012	0.952	0.433	0.254	0.190
3	0.045	0.077	0.011	0.755	0.454	0.214	0.123
4A	0.141	0.201	0.032	1.000	1.000	0.428	0.274
4B	0.146	0.207	0.033	1.000	1.000	0.431	0.327
4C	0.161	0.210	0.043	0.913	1.000	0.403	0.304
5A	0.202	0.247	0.053	1.000	1.000	1.000	0.440
5B	0.197	0.240	0.053	1.000	1.000	1.000	0.442
6	0.069	0.125	0.011	0.852	0.430	0.246	0.180
7A	0.190	0.228	0.056	1.000	1.000	1.000	0.427
7B	0.171	0.212	0.053	1.000	1.000	1.000	0.408
Edge-permuted (ineffective management)							
1	0.071	0.129	0.012	0.917	0.436	0.249	0.183
2	0.071	0.128	0.012	0.929	0.428	0.248	0.186
3	0.056	0.100	0.011	0.819	0.427	0.235	0.152
4A	0.137	0.195	0.032	1.000	1.000	0.434	0.275
4B	0.147	0.205	0.033	1.000	1.000	0.438	0.338
4C	0.155	0.202	0.043	1.000	1.000	0.422	0.310
5A	0.197	0.243	0.053	1.000	1.000	1.000	0.440
5B	0.195	0.237	0.053	1.000	1.000	1.000	0.445
6	0.068	0.124	0.011	0.875	0.435	0.250	0.182
7A	0.192	0.232	0.056	1.000	1.000	1.000	0.436
7B	0.167	0.207	0.053	1.000	1.000	1.000	0.404
Mean-field (ineffective management)							

	Outbreak size - proportion flock infected				Probability of percentage infected sheep		
**Network**	**Mean**	**SD**	**Min**	**Max**	**≥2%**	**≥5%**	**≥10%**
1	0.073	0.131	0.012	0.929	0.428	0.253	0.191
2	0.073	0.132	0.012	0.929	0.429	0.253	0.191
3	0.080	0.144	0.011	0.936	0.457	0.274	0.203
4A	0.142	0.201	0.032	1.000	1.000	0.433	0.279
4B	0.147	0.206	0.033	1.000	1.000	0.432	0.330
4C	0.159	0.208	0.043	1.000	1.000	0.427	0.315
5A	0.202	0.248	0.053	1.000	1.000	1.000	0.442
5B	0.196	0.240	0.053	1.000	1.000	1.000	0.447
6	0.068	0.124	0.011	0.909	0.434	0.249	0.183
7A	0.196	0.238	0.056	1.000	1.000	1.000	0.429
7B	0.171	0.210	0.053	1.000	1.000	1.000	0.420
Observed (active management)							
1	0.014	0.008	0.012	0.214	0.086	0.007	0.001
2	0.014	0.008	0.012	0.274	0.082	0.006	0.002
3	0.012	0.006	0.011	0.191	0.089	0.005	0.001
4A	0.037	0.020	0.032	0.581	1.000	0.082	0.011
4B	0.038	0.020	0.033	0.467	1.000	0.082	0.027
4C	0.049	0.025	0.043	0.478	1.000	0.073	0.024
5A	0.060	0.032	0.053	0.579	1.000	1.000	0.090
5B	0.060	0.031	0.053	0.737	1.000	1.000	0.089
6	0.013	0.006	0.011	0.125	0.076	0.005	0.000
7A	0.063	0.030	0.056	0.722	1.000	1.000	0.083
7B	0.059	0.028	0.053	0.684	1.000	1.000	0.079
Edge-permuted (active management)							
1	0.014	0.007	0.012	0.155	0.083	0.006	0.001
2	0.013	0.007	0.012	0.179	0.080	0.005	0.001
3	0.012	0.007	0.011	0.191	0.083	0.007	0.001

	Outbreak size - proportion flock infected				Probability of percentage infected sheep		
Network	Mean	SD	Min	Max	≥2%	≥5%	≥10%
4A	0.037	0.021	0.032	0.613	1.000	0.082	0.012
4B	0.038	0.022	0.033	0.533	1.000	0.084	0.028
4C	0.049	0.023	0.043	0.609	1.000	0.080	0.023
5A	0.060	0.031	0.053	0.579	1.000	1.000	0.087
5B	0.060	0.030	0.053	0.579	1.000	1.000	0.089
6	0.013	0.007	0.011	0.205	0.076	0.006	0.001
7A	0.062	0.028	0.056	0.611	1.000	1.000	0.080
7B	0.059	0.027	0.053	0.579	1.000	1.000	0.077
Mean-field (active management)							
1	0.013	0.007	0.012	0.262	0.082	0.005	0.001
2	0.014	0.008	0.012	0.262	0.081	0.006	0.001
3	0.012	0.008	0.011	0.287	0.082	0.008	0.001
4A	0.037	0.019	0.032	0.548	1.000	0.086	0.011
4B	0.038	0.019	0.033	0.367	1.000	0.086	0.026
4C	0.049	0.023	0.043	0.478	1.000	0.078	0.022
5A	0.060	0.031	0.053	0.684	1.000	1.000	0.082
5B	0.060	0.031	0.053	0.684	1.000	1.000	0.081
6	0.013	0.008	0.011	0.284	0.079	0.006	0.002
7A	0.063	0.033	0.056	0.833	1.000	1.000	0.082
7B	0.059	0.027	0.053	0.526	1.000	1.000	0.075

1. Mean = mean outbreak size, SD = standard deviation of outbreak sizes, min = minimum, max = maximum

Table S4. Spearman's partial correlation coefficient for model parameters with the final outbreak size in the SEIR models

	Transmission (β)		Recovery (α)		Latent to infectious (Σ)	
Network	Cor_{Sp}	P-value	Cor_{Sp}	P-value	Cor_{Sp}	P-value

**Observed – 28-100 day recovery**						
1	0.416	<0.001	-0.205	<0.001	0.033	0.001
2	0.413	<0.001	-0.210	<0.001	0.029	0.004
3	0.391	<0.001	-0.204	<0.001	0.039	0.000
4A	0.399	<0.001	-0.204	<0.001	0.059	0.000
4B	0.394	<0.001	-0.204	<0.001	0.033	0.001
4C	0.389	<0.001	-0.203	<0.001	0.033	0.001
5A	0.416	<0.001	-0.220	<0.001	0.019	0.052
5B	0.417	<0.001	-0.212	<0.001	0.040	<0.001
6	0.387	<0.001	-0.224	<0.001	0.041	<0.001
7A	0.396	<0.001	-0.198	<0.001	0.036	<0.001
7B	0.376	<0.001	-0.192	<0.001	0.021	0.033
**Observed – 3-15 day recovery**						
1	0.156	<0.001	-0.170	<0.001	0.048	<0.001
2	0.151	<0.001	-0.154	<0.001	0.046	<0.001
3	0.163	<0.001	-0.171	<0.001	0.041	<0.001
4A	0.165	<0.001	-0.158	<0.001	0.039	<0.001
4B	0.146	<0.001	-0.175	<0.001	0.049	<0.001
4C	0.152	<0.001	-0.158	<0.001	0.050	<0.001
5A	0.141	<0.001	-0.171	<0.001	0.039	<0.001
5B	0.165	<0.001	-0.149	<0.001	0.044	<0.001
6	0.146	<0.001	-0.147	<0.001	0.033	0.001
7A	0.145	<0.001	-0.164	<0.001	0.038	<0.001
7B	0.141	<0.001	-0.151	<0.001	0.035	<0.001

1. Cor_{SP} = Spearman's partial correlation coefficient
